# Hyperpolarized ^13^C MRI of Tumor Metabolism Demonstrates Early Metabolic Response to Neoadjuvant Chemotherapy in Breast Cancer

**DOI:** 10.1148/rycan.2020200017

**Published:** 2020-07-31

**Authors:** Ramona Woitek, Mary A. McLean, Andrew B. Gill, James T. Grist, Elena Provenzano, Andrew J. Patterson, Stephan Ursprung, Turid Torheim, Fulvio Zaccagna, Matthew Locke, Marie-Christine Laurent, Sarah Hilborne, Amy Frary, Lucian Beer, Leonardo Rundo, Ilse Patterson, Rhys Slough, Justine Kane, Heather Biggs, Emma Harrison, Titus Lanz, Bristi Basu, Richard Baird, Evis Sala, Martin J. Graves, Fiona J. Gilbert, Jean E. Abraham, Carlos Caldas, Kevin M. Brindle, Ferdia A. Gallagher

**Affiliations:** From the Departments of Radiology (R.W., A.B.G., J.T.G., A.J.P., S.U., F.Z., M.L., M.C.L., S.H., A.F., L.B., L.R., E.S., M.J.G., F.J.G., F.A.G.), Oncology (J.K., H.B., E.H., B.B., R.B., J.E.A., C.C.), and Biochemistry (K.M.B.), the Cambridge Breast Cancer Research Unit (E.P., J.K., H.B., E.H., R.B., J.E.A., C.C.), University of Cambridge, Cambridge, England; Departments of Radiology (A.J.P., I.P., R.S., M.J.G., F.J.G., F.A.G.) and Histopathology (E.P.), Addenbrooke’s Hospital, Cambridge University Hospitals NHS Foundation Trust, Cambridge, England; Cancer Research UK Cambridge Centre, Cambridge, England (R.W., M.A.M., E.P., T.T., L.B., L.R., E.S., J.E.A., C.C., K.M.B., F.A.G.); Department of Biomedical Imaging and Image-guided Therapy, Medical University of Vienna, Waehringer Guertel 18-20, Vienna 1090, Austria (R.W., L.B.); Cancer Research UK Cambridge Institute, University of Cambridge, Li Ka Shing Centre, Cambridge, England (M.A.M., T.T., C.C., K.M.B.); and RAPID Biomedical, Rimpar, Germany (T.L.).

## Abstract

**Purpose:**

To compare hyperpolarized carbon 13 (^13^C) MRI with dynamic contrast material–enhanced (DCE) MRI in the detection of early treatment response in breast cancer.

**Materials and Methods:**

In this institutional review board–approved prospective study, a woman with triple-negative breast cancer (age, 49 years) underwent ^13^C MRI after injection of hyperpolarized [1–carbon 13 {^13^C}]-pyruvate and DCE MRI at 3 T at baseline and after one cycle of neoadjuvant therapy. The ^13^C-labeled lactate-to-pyruvate ratio derived from hyperpolarized ^13^C MRI and the pharmacokinetic parameters transfer constant (*K*^trans^) and washout parameter (*k*_ep_) derived from DCE MRI were compared before and after treatment.

**Results:**

Exchange of the ^13^C label between injected hyperpolarized [1-^13^C]-pyruvate and the endogenous lactate pool was observed, catalyzed by the enzyme lactate dehydrogenase. After one cycle of neoadjuvant chemotherapy, a 34% reduction in the ^13^C-labeled lactate-to-pyruvate ratio resulted in correct identification of the patient as a responder to therapy, which was subsequently confirmed via a complete pathologic response. However, DCE MRI showed an increase in mean *K*^trans^ (132%) and mean *k*_ep_ (31%), which could be incorrectly interpreted as a poor response to treatment.

**Conclusion:**

Hyperpolarized ^13^C MRI enabled successful identification of breast cancer response after one cycle of neoadjuvant chemotherapy and may improve response prediction when used in conjunction with multiparametric proton MRI.

**Keywords:** Breast, MR-Spectroscopy, Molecular Imaging-Cancer, Molecular Imaging-Clinical Translation, Neoplasms-Primary, Oncology, Tumor Response

Published under a CC BY 4.0 license.

**Summary**

Early response assessment in a patient with breast cancer undergoing neoadjuvant chemotherapy is feasible using hyperpolarized carbon 13 MRI.

**Key Points**

■ To the authors’ knowledge, this is the first reported use of hyperpolarized carbon 13 (^13^C) MRI to detect an early metabolic response to neoadjuvant chemotherapy in a patient with breast cancer, demonstrating a 34% decrease in ^13^C lactate labeling after one cycle of therapy.
■ This finding was supported by a decrease in tumor volume of 76% and was confirmed as pathologic complete response at surgery; however, pharmacokinetic parameters derived from dynamic contrast-enhanced MRI showed an increase in the mean transfer constant (132%) and mean washout parameter (31%), which are typically associated with nonpathologic complete response.

## Introduction

Breast cancer is the most common cancer in women, accounting for over 2 million new cases annually worldwide (1). Triple-negative breast cancer (TNBC) represents 15%–20% of all breast cancers and is defined by low or absent expression of hormone receptors and lack of amplification or overexpression of human epidermal growth factor receptor type 2 (HER2). Therefore, TNBC lacks any established targeted treatment options, such as endocrine or anti-HER2 therapy. Compared with other subtypes of breast cancer, TNBC has an adverse overall outcome, but it often shows good response to neoadjuvant chemotherapy (2). In contrast to adjuvant chemotherapy, neoadjuvant chemotherapy allows assessment of treatment response in situ and downstaging of the tumor prior to surgery (2). Furthermore, complete pathologic response after neoadjuvant chemotherapy is an important prognostic factor in patients with TNBC, as it is indicative of longer event-free and overall survival (3,4).

Early prediction of pathologic complete response at imaging would assist patient care and has been demonstrated using several approaches, including multiparametric proton MRI, but it remains challenging owing to the low accuracy of these techniques (5,6). The delayed identification of nonresponders results in increased patient morbidity from side effects as well as a risk of metastases from chemoresistant cells; it also has substantial economic implications (7).

TNBC frequently shows metabolic changes, including a switch to glycolysis, resulting in increased production of lactate, either secondary to hypoxia or as a consequence of aerobic glycolysis, which is known as the Warburg effect (8). Hyperpolarized carbon 13 (^13^C) MRI is an emerging clinical technique that allows dynamic imaging of metabolic reactions in vivo, such as ^13^C label exchange between pyruvate and lactate after intravenous injection of hyperpolarized [1–carbon 13 {^13^C}]-pyruvate (9). Preclinical studies of treatment response have shown a decrease in hyperpolarized ^13^C label exchange between pyruvate and lactate as early as 24 hours after cytotoxic treatment in a range of cancer models, including breast cancer (10,11). A study has recently demonstrated the feasibility of this technique for use in the assessment of patients with breast cancer, showing higher levels of lactate labeling in higher-grade tumors, including all the TNBCs assessed (12). The first clinical example of response assessment using hyperpolarized ^13^C MRI showed a decreased hyperpolarized lactate signal after 6 weeks of androgen deprivation therapy in a patient with prostate cancer (13).

To our knowledge, this is the first report to demonstrate the use of hyperpolarized ^13^C MRI to monitor early response to neoadjuvant chemotherapy in human breast cancer, and here we have compared this with dynamic contrast material–enhanced (DCE) MRI. In this study, we show that hyperpolarized ^13^C MRI is complementary to conventional hydrogen 1 (^1^H) MRI in breast cancer, and our findings support the use of this technique as part of larger clinical studies in the future.

## Materials and Methods

### Study Design

This prospective study protocol had institutional review board (Cambridge South Research Ethics Committee) approval, and written informed consent was obtained. MRI of both breasts of a 49-year-old woman was performed in 2018, 1 day before the start of neoadjuvant chemotherapy (baseline) and after a 3-week cycle of chemotherapy (follow-up) with a clinical 3-T scanner (MR750; GE Healthcare, Waukesha, Wis).

### ^1^H MRI

Diagnostic quality ^1^H MRI of the breast was performed using a dedicated eight-channel phased-array receive-only ^1^H breast coil with the patient in the prone position and a three-dimensional fast spoiled gradient-echo sequence with k-space data sharing for DCE MRI (volume image breast assessment–time-resolved imaging of contrast kinetics [VIBRANT-TRICKS]), as previously described (14). Images were acquired with a repetition time of 7.1 msec, echo time of 3.8 msec, an in-plane voxel size of 0.68 × 0.68 mm, a slice thickness of 1.4 mm, a field of view of 350 mm, a matrix of 512 × 512, spectral-spatial water excitation, and a flip angle of 12°. Forty-eight VIBRANT-TRICKS volumes were acquired over 8 minutes, with a temporal resolution of 9.4 seconds. Contrast agent injection was started between volumes two and three. Gadobutrol (Gadovist; Bayer Healthcare, Berlin, Germany) was injected at 0.1 mmol per kilogram of body weight and a flow rate of 3.0 mL/sec followed by a 25-mL saline flush.

Tumor volumes of interest were drawn manually on the DCE MRI data by an attending radiologist (R.W.) with 10 years of radiologic experience who specialized in breast imaging (3D Slicer; *https://www.slicer.org*) (15). For the baseline study, two sets of volumes of interest were drawn, one covering the entire tumor and a second excluding the central tumor area showing low or delayed enhancement. These volumes of interest were used to calculate tumor volumes in 3D Slicer and to extract voxelwise pharmacokinetic parameters (transfer constant [*K*^trans^], washout parameter [*k*_*ep*_], extravascular extracellular volume [*v*_*e*_], and area under the contrast concentration versus time curve 90 seconds after contrast material injection [iAUC90]) (MIStar; Apollo Medical Imaging Technology, Melbourne, Australia). Only voxels with a high enough goodness of fit (*r*^*2*^ ≥ 0.75) were included in the analyses. Mean values of the pharmacokinetic parameters were calculated for all sets of volumes of interest.

### ^13^C MRI and Postprocessing

Pharmacy kits and samples containing 1.47 g of [1-^13^C]-pyruvic acid and 15 mmol/L of an electron paramagnetic agent were prepared, hyperpolarized, and rapidly dissolved using 38 mL of superheated sterile water, and pharmaceutical quality and suitability for injection were confirmed after filtration of the electron paramagnetic agent to less than or equal to 3 µM, as described previously (16). Hyperpolarized pyruvate solution (0.4 mL/kg at a concentration of approximately 250 mmol/L) was injected at a rate of 5 mL/sec followed by a 25-mL saline flush. For ^13^C MRI, a dedicated eight-channel ^13^C breast coil (RAPID Biomedical, Rimpar, Germany) was used with a phantom containing a ^13^C-labeled 8 mol/L urea sample (Sigma-Aldrich, St Louis, Mo) positioned adjacent to the tumor-containing breast. Images were acquired using a dynamic coronal iterative decomposition with echo asymmetry and least-squares estimation (known as IDEAL) spiral chemical shift imaging sequence (17). Spectral data from the eight breast coil channels were summed over time. Complex imaging data from the eight breast coil channels were summed over the time series, then combined as the sum of squares with signal from each channel weighted by the maximal signal-to-noise ratio (SNR) of pyruvate. Pyruvate, pyruvate hydrate, lactate, alanine, and bicarbonate images were reconstructed. Tumor regions of interest were generated by thresholding the sum of the summed lactate and pyruvate signals using custom software developed in MATLAB (version 2019b, MathWorks, Natick, Mass) so that the diameter of the region of interest on the ^13^C images matched the maximum transverse tumor diameter on the DCE MRI scans at peak enhancement. Since the noise distribution on images of different individual metabolites is the same, noise was characterized from an entire image in which spiral acquisition artifacts were absent. We used the following equation to generate the SNR for pyruvate and lactate (SNR_metabolite_), on which further calculations of metabolite ratios were based:
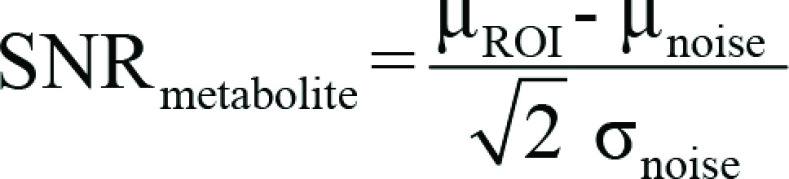
where μ_ROI_ is the mean signal intensity in the tumor region of interest, and μ_noise_ and σ_noise_ are the mean and standard deviation, respectively, of the noise signal and both were computed from the entire noise image series, as described previously.

By dividing the SNR of lactate summed over the entire image time course by the summed pyruvate SNR, the ^13^C-labeled lactate-to-pyruvate (Lac/Pyr) ratio was calculated. The apparent exchange rate constant for pyruvate-lactate exchange (*k*_PL_) was computed based on a frequency-domain approach and linear least-squares fitting using a two-site exchange model (18).

The lactate and pyruvate SNR and the Lac/Pyr ratio in this patient at baseline were included in a previous feasibility study (12).

## Results

We report the case of a woman with TNBC undergoing neoadjuvant chemotherapy and early response assessment, who underwent assessment before and after the first cycle of treatment with multinuclear MRI of the breast. The patient was a 49-year-old woman with unifocal, grade 3, triple-negative (estrogen receptor weakly positive [Allred score, 3; 5% positive cells], progesterone receptor-negative, human epidermal growth factor receptor type 2–negative [score, 1+]) invasive carcinoma of no specific type in the right breast (upper inner quadrant) and a Ki67 result of 90% positive cells with no necrosis at biopsy. Axillary lymph node core biopsy results were negative for malignancy. CT scans of the chest, abdomen, and pelvis were negative for metastatic disease. No family history of breast cancer was reported. The patient was negative for germline *BRCA1* and *BRCA2* mutations.

Baseline multinuclear (^1^H and ^13^C) MRI was performed 1 day prior to treatment and revealed a unifocal cancer measuring 34 × 35 × 38 mm in the posterior aspect of the right breast. The tumor showed avid contrast enhancement peripherally, with delayed enhancement centrally, which was most likely fibrotic in nature, as the signal intensity on T2-weighted images was low centrally (Fig 1). After one cycle of neoadjuvant chemotherapy (three weekly doses of paclitaxel [Taxol; Bristol-Myers-Squibb, Princeton, NJ] and one dose of carboplatin), multinuclear MRI was repeated (Figs 1, 2) and imaging data showed a decrease in the Lac/Pyr signal intensity ratio of 34%, with a similar decrease of 37% in *k*_PL_ for the exchange of hyperpolarized ^13^C label between pyruvate and lactate. This was accompanied by a decrease in tumor volume of 76%, all of which demonstrated early signs of successful treatment response. In contrast, DCE MRI showed an increase in mean *K*^trans^ of 132% and a smaller increase in mean *k*_ep_ of 31% when compared with evaluation of the entire tumor at baseline (Fig 3); similar results were shown when the central delayed enhancing area was excluded, with a 113% increase in *K*^trans^ and a 23% increase in *k*_ep_.

**Figure 1a:**
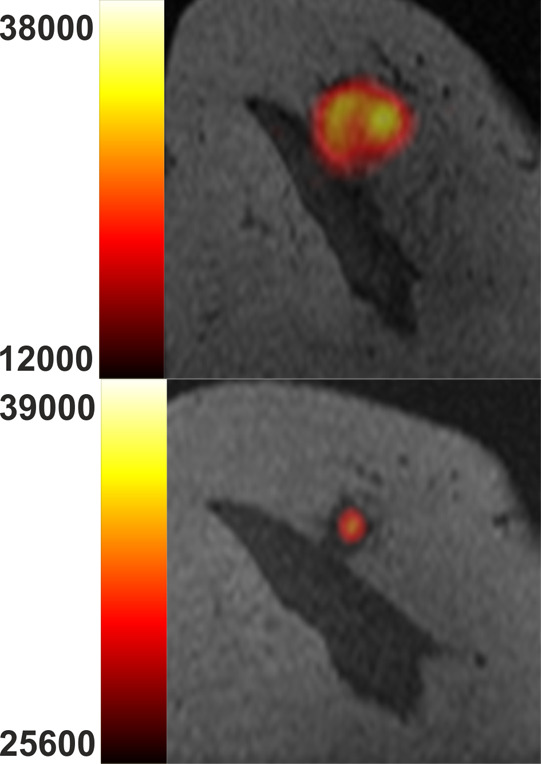
Multinuclear hydrogen 1 and ^13^C MR images of the right breast at baseline (top) and after one cycle of neoadjuvant chemotherapy (bottom). **(a)** Coronal summed hyperpolarized [1-^13^C]-pyruvate and **(b)** [1-^13^C]-lactate signal overlaid on unenhanced T1-weighted images. **(c)** Coronal dynamic contrast-enhanced MR image obtained 150 seconds after contrast agent injection and **(d)** overlaid transfer constant (*K*^trans^) map.

**Figure 1b:**
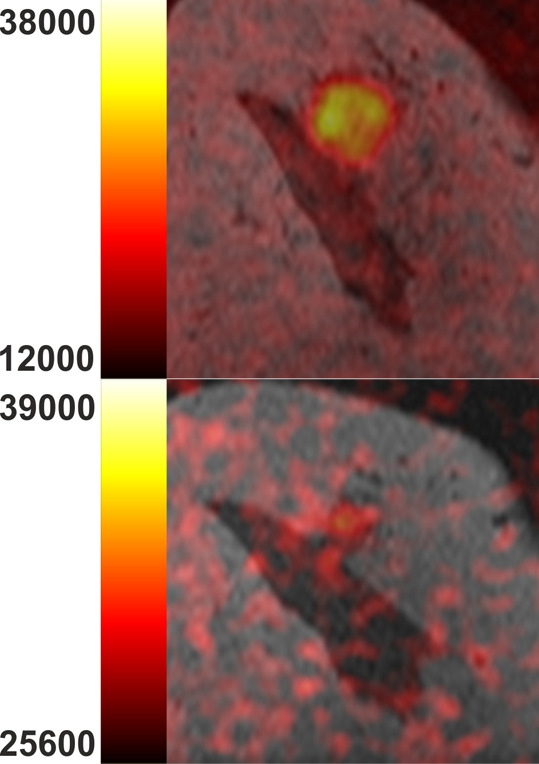
Multinuclear hydrogen 1 and ^13^C MR images of the right breast at baseline (top) and after one cycle of neoadjuvant chemotherapy (bottom). **(a)** Coronal summed hyperpolarized [1-^13^C]-pyruvate and **(b)** [1-^13^C]-lactate signal overlaid on unenhanced T1-weighted images. **(c)** Coronal dynamic contrast-enhanced MR image obtained 150 seconds after contrast agent injection and **(d)** overlaid transfer constant (*K*^trans^) map.

**Figure 1c:**
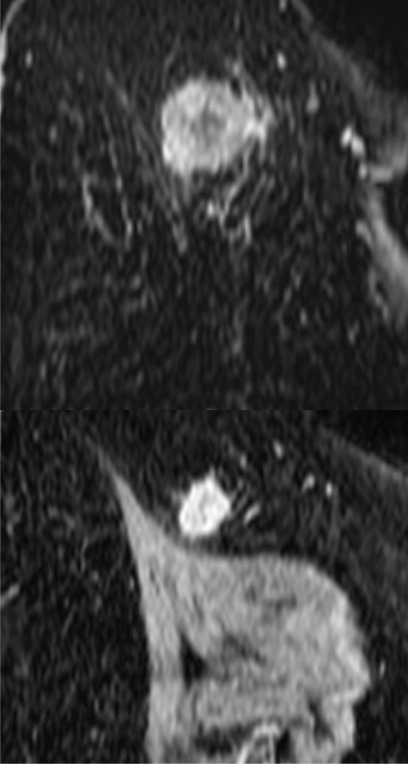
Multinuclear hydrogen 1 and ^13^C MR images of the right breast at baseline (top) and after one cycle of neoadjuvant chemotherapy (bottom). **(a)** Coronal summed hyperpolarized [1-^13^C]-pyruvate and **(b)** [1-^13^C]-lactate signal overlaid on unenhanced T1-weighted images. **(c)** Coronal dynamic contrast-enhanced MR image obtained 150 seconds after contrast agent injection and **(d)** overlaid transfer constant (*K*^trans^) map.

**Figure 1d:**
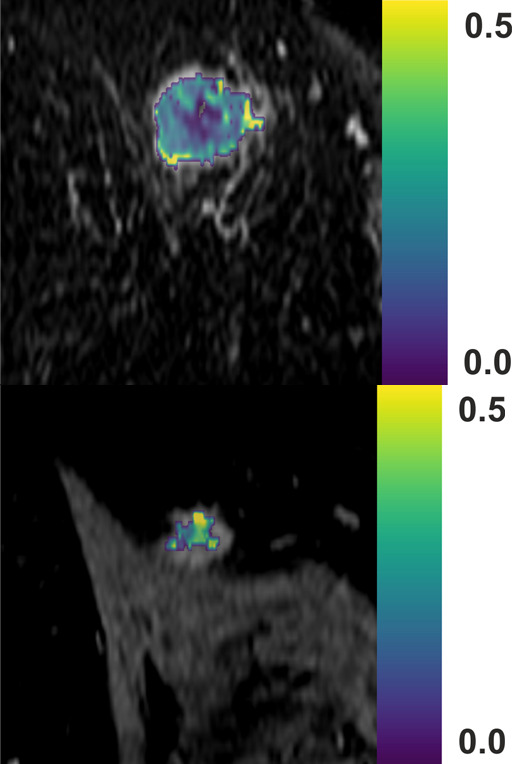
Multinuclear hydrogen 1 and ^13^C MR images of the right breast at baseline (top) and after one cycle of neoadjuvant chemotherapy (bottom). **(a)** Coronal summed hyperpolarized [1-^13^C]-pyruvate and **(b)** [1-^13^C]-lactate signal overlaid on unenhanced T1-weighted images. **(c)** Coronal dynamic contrast-enhanced MR image obtained 150 seconds after contrast agent injection and **(d)** overlaid transfer constant (*K*^trans^) map.

**Figure 2a:**
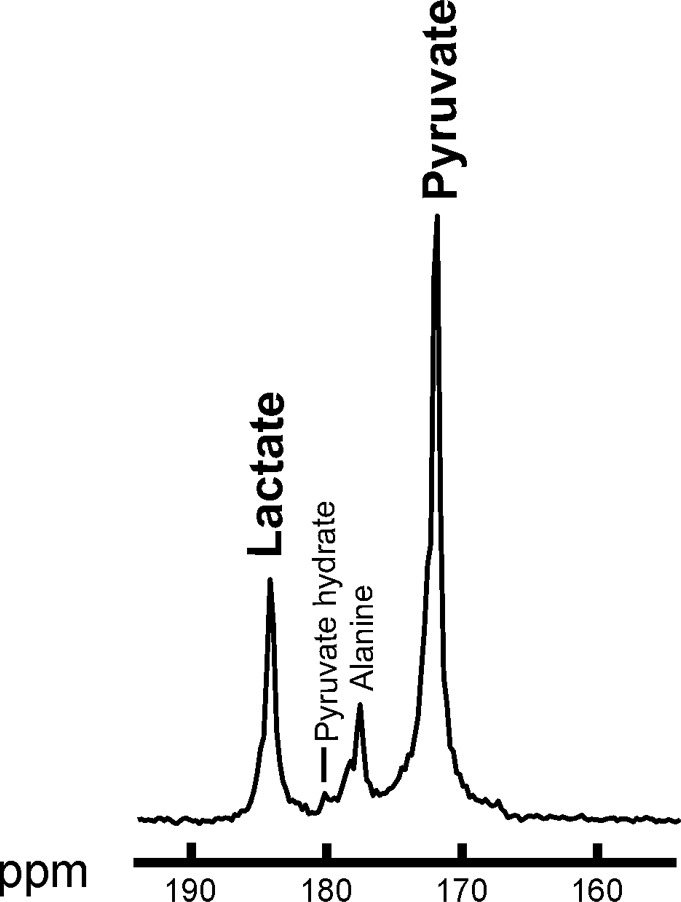
Summed ^13^C spectra over time after ^13^C-pyruvate bolus arrival in the breast. Summed spectra for **(a)** baseline and **(b)** after one cycle of neoadjuvant chemotherapy. ppm = parts per million.

**Figure 2b:**
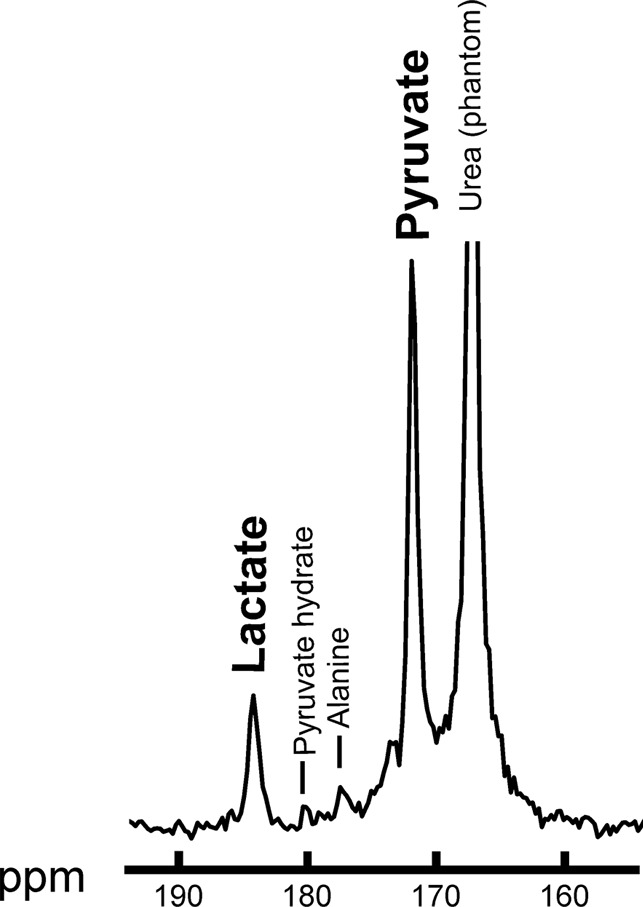
Summed ^13^C spectra over time after ^13^C-pyruvate bolus arrival in the breast. Summed spectra for **(a)** baseline and **(b)** after one cycle of neoadjuvant chemotherapy. ppm = parts per million.

**Figure 3:**
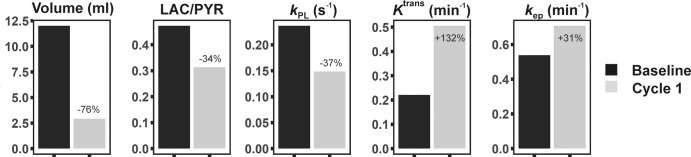
Changes in volume, ^13^C-lactate-to-pyruvate (Lac/Pyr) ratio, exchange rate constant (*k*_PL_), transfer constant (*K*^trans^), and washout parameter (*k*_ep_) between baseline and follow-up imaging after one cycle (cycle 1) of neoadjuvant chemotherapy. While tumor volume and Lac/Pyr ratio decreased during treatment in this responding patient, pharmacokinetic parameters *K*^trans^ and *k*_ep_ increased. Changes in Lac/Pyr ratio and *k*_PL_ are based on imaging data, not spectra. The ^13^C MRI-based metrics were therefore more reliable than dynamic contrast material–enhanced MRI in correctly identifying this patient as a responder. Volumes of interest covering the entire tumor at the baseline and follow-up imaging were used to calculate these mean values.^.^

After seven cycles of neoadjuvant chemotherapy (four cycles of weekly paclitaxel and with carboplatin every 3 weeks, followed by three cycles of epirubicin and cyclophosphamide), the patient underwent wide local excision of the cancer. At histopathologic examination, no residual invasive or in situ carcinoma was identified, in keeping with the pathologic complete response.

## Discussion

Identification of early response to neoadjuvant chemotherapy in patients with breast cancer is challenging with currently available imaging methods (5,6). Pharmacokinetic modeling of DCE MRI can improve early identification of nonresponders to neoadjuvant chemotherapy when compared with measurements of tumor size (19), although some controversy persists about the exact role of the DCE in- and outflow parameters (*K*^trans^ and *k*_ep_) in stratifying response. A decrease in these constants during neoadjuvant chemotherapy is frequently associated with pathologic complete response (20,21). However, here we observed that both *K*^trans^ and *k*_ep_ were increased markedly after one cycle of treatment (by 132% and 32%, respectively), which would have incorrectly identified this patient as a nonresponder. This finding is in keeping with previous work, which has demonstrated the poor sensitivity of pharmacokinetic parameters in the early identification of pathologic complete response despite high specificity (20). For example, in a previous study, responders with early tumor shrinkage after one cycle of neoadjuvant chemotherapy showed an increased *K*^trans^ in 22% of cases and an increased *k*_ep_ in 11% of cases on early DCE MRI follow-up (22). DCE MRI performed with high spatial and temporal resolution to allow pharmacokinetic modeling is feasible and can be integrated into routine clinical scanning protocols to allow quantitative data analysis in addition to kinetic maps used routinely for radiologic assessment of breast tumors.

Preclinical hyperpolarized ^13^C MRI studies investigating treatment response have shown a significant decrease in ^13^C label exchange between ^13^C pyruvate and ^13^C lactate as early as 24–48 hours after cytotoxic treatment in murine lymphoma and breast cancer models (10,11). An initial clinical report on androgen deprivation therapy for prostate cancer in one patient showed decreased ^13^C label exchange between pyruvate and lactate 6 weeks after initiation of treatment (13). The feasibility of using this method in patients with breast cancer has been demonstrated recently (12): Higher Lac/Pyr ratios were observed in larger and more aggressive tumors (including all triple-negative cancers), and this correlated with the expression of the plasma membrane transporter mediating uptake of pyruvate into tumor cells (monocarboxylate transporter 1) and hypoxia-inducible factor 1-α. We have shown here that hyperpolarized ^13^C MRI can be used to detect early response to neoadjuvant chemotherapy in a patient with breast cancer. Metabolic response was demonstrated after one cycle of neoadjuvant chemotherapy by decreases in the Lac/Pyr ratio and *k*_PL_ of 34% and 37%, respectively. These findings were supported by a concurrent decrease in tumor volume of 76% and were eventually confirmed as pathologic complete response at surgery. Hyperpolarized ^13^C MRI may therefore improve the prediction of response when used in conjunction with conventional multiparametric MRI.

Although glycolysis can be probed indirectly with other techniques, such as PET and conventional ^1^H MR spectroscopy, hyperpolarized ^13^C MRI offers a number of potential advantages. The technique is free of ionizing radiation, does not require a long uptake time (as is the case with fluorine 18 [^18^F] fluorodeoxyglucose [FDG]), and, because it depends on lactate pool size, it assesses more of the glycolytic pathway than ^18^F FDG PET, which effectively assesses the first two steps of glycolysis. A preclinical study recently demonstrated the superiority of hyperpolarized ^13^C MRI over ^18^F FDG PET in early response assessment in a breast cancer model (23). Lactate measurements using ^1^H MR spectroscopy remain challenging in patients with breast cancer owing to the high abundance of lipids in breast tumors and surrounding fat tissue (24). Other multinuclear MRI approaches have been shown to complement ^1^H MR spectroscopy in detecting response to treatment in breast cancer (25,26).

The limitations of our study were that the results are from one patient, and the response was assessed after a full cycle of chemotherapy, when there was already a decrease in tumor volume. Further studies are needed to assess whether the metabolic changes observed in this study precede changes in tumor volume, as has been demonstrated preclinically (23).

In conclusion, we showed that early response assessment in breast cancer using hyperpolarized ^13^C MRI is feasible. Our results also support a potential clinical role for the technique in conjunction with multiparametric breast MRI by enabling an early readout of response that aids clinical decision making and may facilitate the development of targeted drugs for the treatment of TNBC.
